# Discussion on the thermal conductivity enhancement of nanofluids

**DOI:** 10.1186/1556-276X-6-124

**Published:** 2011-02-09

**Authors:** Huaqing Xie, Wei Yu, Yang Li, Lifei Chen

**Affiliations:** 1 School of Urban Development and Environmental Engineering, Shanghai Second Polytechnic University, Shanghai 201209, China

## Abstract

Increasing interests have been paid to nanofluids because of the intriguing heat transfer enhancement performances presented by this kind of promising heat transfer media. We produced a series of nanofluids and measured their thermal conductivities. In this article, we discussed the measurements and the enhancements of the thermal conductivity of a variety of nanofluids. The base fluids used included those that are most employed heat transfer fluids, such as deionized water (DW), ethylene glycol (EG), glycerol, silicone oil, and the binary mixture of DW and EG. Various nanoparticles (NPs) involving Al_2_O_3 _NPs with different sizes, SiC NPs with different shapes, MgO NPs, ZnO NPs, SiO_2 _NPs, Fe_3_O_4 _NPs, TiO_2 _NPs, diamond NPs, and carbon nanotubes with different pretreatments were used as additives. Our findings demonstrated that the thermal conductivity enhancements of nanofluids could be influenced by multi-faceted factors including the volume fraction of the dispersed NPs, the tested temperature, the thermal conductivity of the base fluid, the size of the dispersed NPs, the pretreatment process, and the additives of the fluids. The thermal transport mechanisms in nanofluids were further discussed, and the promising approaches for optimizing the thermal conductivity of nanofluids have been proposed.

## Introduction

More efficient heat transfer systems are increasingly preferred because of the accelerating miniaturization, on the one hand, and the ever-increasing heat flux, on the other. In many industrial processes, including power generation, chemical processes, heating or cooling processes, and microelectronics, heat transfer fluids such as water, mineral oil, and ethylene glycol always play vital roles. The poor heat transfer properties of these common fluids compared to most solids is a primary obstacle to the high compactness and effectiveness of heat exchangers [1]. An innovative way of improving the thermal conductivities of working media is to suspend ultrafine metallic or nonmetallic solid powders in traditional fluids since the thermal conductivities of most solid materials are higher than those of liquids. A novel kind of heat transfer enhancement fluid, the so-called nanofluid, has been proposed to meet the demands [2].

"Nanofluid" is an eye-catching word in the heat transfer community nowadays. The thermal properties, including thermal conductivity, viscosity, specific heat, convective heat transfer coefficient, and critical heat flux have been studied extensively. Several elaborate and comprehensive review articles and books have addressed thermal transport properties of nanofluids [1,3-6]. Among all these properties, thermal conductivity is the first referred one, and it is believed to be the most important parameter responsible for the enhanced heat transfer. Investigations on the thermal conductivity of nanofluids have been drawing the greatest attention of the researchers. A variety of physical and chemical factors, including the volume fraction, the size, the shape, and the species of the nanoparticles (NPs), pH value and temperature of the fluids, the Brownian motion of the NPs, and the aggregation of the NPs, have been proposed to play their respective roles on the heat transfer characteristics of nanofluids [7-19]. Extensive efforts have been made to improve the thermal conductivity of nanofluids [7-19] and to elucidate the thermal transport mechanisms in nanofluids [20-23].

The authors have carried out a series of studies on the heat transfer enhancement performance of nanofluids. A variety of nanofluids have been produced by the one- or two-step method. The base fluids used include deionized water (DW), ethylene glycol (EG), glycerol, silicone oil, and the binary mixture of DW and EG (DW-EG). Al_2_O_3 _NPs with different sizes, SiC NPs with different shapes, MgO NPs, ZnO NPs, SiO_2 _NPs, Fe_3_O_4 _NPs, TiO_2 _NPs, diamond NPs (DNPs), and carbon nanotubes (CNTs) with different pretreatments have been used as additives. The thermal conductivities of these nanofluids have been measured by transient hot wire (THW) method or short hot wire (SHW) technique. In this article, the experimental results that elucidate the influencing factors for thermal conductivity enhancement of nanofluids are presented. The thermal transport mechanisms in nanofluids and promising approaches for optimizing the thermal conductivity of nanofluids are further presented.

## Preparation of nanofluids

Two techniques have been applied to prepare nanofluids in our studies: two- and one-step techniques. Most of the studied nanofluids were prepared by the two-step technique. During the procedure of two-step technique, the dispersed NPs were prepared by chemical or physical methods first, and then the NPs were added into a specified base fluid, with or without pretreatment and surfactant based on the need. In the preparation of nanofluids containing metallic NPs, one-step technique was employed.

The process was quite simple in the preparation of nanofluids containing oxide NPs like Al_2_O_3_, ZnO, MgO, TiO_2_, and SiO_2 _NPs. The NPs were obtained commercially and were dispersed into a base fluid in a mixing container. The NPs were deagglomerated by intensive ultrasonication after being mixed with the base fluid, and then the suspensions were homogenized by magnetic force agitation.

Two-step method was used to prepare graphene nanofluids. The first step was to prepare graphene nanosheets. Functionalized graphene was gained through a modified Hummers method as described elsewhere [24]. Graphene nanosheets were obtained by exfoliation of graphite in anhydrous ethanol. The product was a loose brown powder, and it had good hydrophilic nature. The graphene nanosheets could be dispersed well in polar solvents, like DW and EG, without the use of surfactant. For liquid paraffin (LP)-based nanofluid, oleylamine was used as the surfactant. The fixed quality of graphene nanosheets with different volume fractions was dispersed in the base fluids.

Severe aggregation always takes place in the as-prepared CNTs (pristine CNTs: PCNTs) because of the non-reactive surfaces, intrinsic Von der Waals forces, and very large specific surface areas, and aspect ratios [25]. In CNT nanofluid preparations, surfactant addition is an effective way to enhance the dispersibility of CNTs [26-28]. However, surfactant molecules attaching on the surfaces of CNTs may enlarge the thermal resistance between the CNTs and the base fluid [29], which limits the enhancement of the effective thermal conductivity. The steps involved in the preparation of surfactant-free CNT nanofluids include (1) disentangling the nanotube entanglement and introducing hydrophilic functional groups on the surfaces of the nanotubes by chemical treatments; (2) cutting the treated CNTs (TCNTs) to optimal length by ball milling; and (3) dispersing the treated and cut CNTs into base fluids. CNTs including single-walled CNTs (SWNTs), double-walled CNTs (DWNTs), and multi-walled CNTs (MWNTs) were obtained commercially. Two chemical routes for treating CNTs were used for this study. One is oxidation with concentrated acid, and the other is mechanochemical reaction with potassium hydroxide (KOH). The detailed treatment processes have been described elsewhere [8,30].

Phase transfer method was used to prepare stable kerosene-based Fe_3_O_4 _magnetic nanofluid. The first step is to synthesize Fe_3_O_4 _NPs in water by coprecipitation. Oleic acid was added to modify the NPs. When kerosene is added to the mixture with slow stirring, the phase transfer process took place spontaneously. There was a distinct phase interface between the aqueous and kerosene. After the removal of the aqueous phase using a pipette, the kerosene-based Fe_3_O_4 _nanofluid was obtained [31].

Nanofluids containing copper NPs were prepared using direct chemical reduction method. Stable nanofluids were obtained with the addition of poly(vinylpyrrolidone) (PVP). The diameters of copper NPs prepared by chemical reduction procedure are in the range of 5-10 nm, and copper NPs disperse well with no clear aggregation [32].

Surface modification is always used to enhance the dispersibility of NPs in the preparation of nanofluids. For example, diamond NPs (DNPs) were purified and surface modified by acid mixtures of perchloric acid, nitric acid and hydrochloric acid according to the literature [33] before being dispersed into the base fluids. SiC NPs were heated in air to remove the excess free carbon and their surfaces modified to enhance their dispersibility.

## Consideration on the thermal conductivity measurement

Inconsistent experimental results and controversial arguments arise unceasingly from different groups conducting research on nanofluids, indicating the complexity of the thermal transport in nanofluids. Through an investigation, a large degree of randomness and scatter have been observed in the experimental data published in the open literature. Given the inconsistency in the data, it is impossible to develop a convincing and comprehensive physical-based model that can predict all the trends. To clarify the suspicion on the scattered and wide-ranging experimental results of the thermal conductivity obtained by different groups, it is preferred to screen the measurement technique and procedure to guarantee the accuracy of the obtained results.

Several researchers observed the "time-dependent characteristic" of thermal conductivity [34-36], that is to say, thermal conductivity was the highest right after nanofluid preparation, and then it decreased considerably with elapsed time. We believe that the "time-dependent characteristic" does not represent the essence of thermal conduction capability of nanofluids. The following two factors may account for this phenomenon. The first one is the motion of the remained particle caused by the agitation during the nanofluid preparation. To make a nanofluid homogeneous and long-term stable, it is always subjected to intensive agitation including magnetic stirring and sonication to destroy the aggregation of the suspended NPs. In very short time after nanofluid preparation, the NPs still keep moving in the base fluid (different from Brownian motion). The motion of the remained particle would cause convection and enhance the energy transport in the nanofluids. Second, when a nanofluid is subjected to long-time sonication, its temperature would be increased. The temperature goes down gradually to the surrounding temperature (thermal conductivity measurement temperature). In very short time after the sonication stops, the process has been remaining. Although the temperature decrease is not severe, the thermal conductivity obtained is very sensitive to the temperature decrease when the transient hot-wire technique is used to measured the thermal conductivity. In our measurements, this phenomenon would be observed. When measuring the thermal conductivity at an unequilibrium state, it was found that the measured data might be very different for a nanofluid even at a specific temperature (see 25°C) if the process to reach this temperature is different. If the temperature is increasing, then the datum obtained of the thermal conductivity would be lower than the true value. While the temperature is decreasing, the datum obtained of the thermal conductivity would be higher than the true value. Therefore, keeping a nanofluid stable and initial equilibrium is very important to obtain accurate thermal conductivity data in measurements.

A transient short hot-wire method was used to measure the thermal conductivities of the base fluids (*k*_0_) and the nanofluids (*k*). The detailed measurement principle, procedure, and error analysis have been described in [37]. In our measurements, a platinum wire with a diameter of 50 μm was used for the hot wire, and it served both as a heating unit and as an electrical resistance thermometer. The platinum wire was coated with an insulation layer of 7-μm thickness. Initially the platinum wire immersed in media was kept at equilibrium with the surroundings. When a regulation voltage was supplied to initiate the measurement, the electrical resistance of the wire changed proportionally with the rise in temperature. The thermal conductivity was calculated from the slope of the rise in the wire's temperature against the logarithmic time interval. The uncertainty of this measurement is estimated to be within ± 1.0%. A temperature-controlled bath was used to maintain different temperatures of the nanofluids. Instead of monitoring the temperature of the bath, a thermocouple was positioned inside the sample to monitor the temperature on the spot. When the temperature of the sample reached a steady value, the authors waited for further 20 min to make sure that the initial state is at equilibrium. At every tested temperature, measurements were made three times and the average values were taken as the final results. A 20-min interval was needed between two successive measurements. After the above-mentioned careful check on the measurement condition and procedure, the authors could gain confidence on the experimental results.

## Influencing factors of thermal conductivity enhancement

In the experiment of the study, it was found that the thermal conductivity enhancements of nanofluids might be influenced by multi-faceted factors including the volume fraction of the dispersed NPs, the tested temperature, the thermal conductivity of the base fluid, the size of the dispersed NPs, the pretreatment process, and the additives of the fluids. The effects of these factors are presented in this section.

### Particle loading

**Figure 1:**
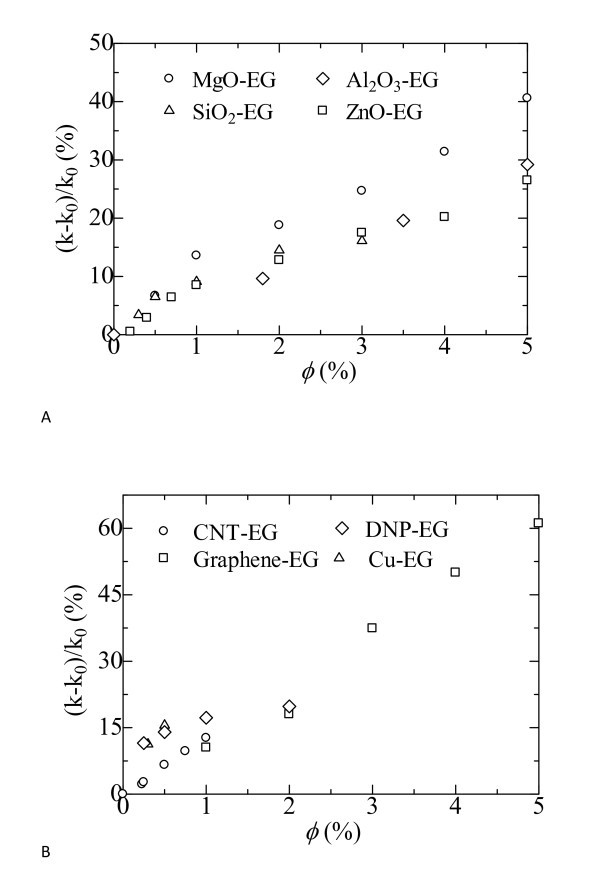
**Thermal conductivity enhancement ratios of the nanofluids as a function of nanoparticle loading**. **(a) **Oxide nanofluids: MgO-EG [38]; Al_2_O_3_-EG [7]; ZnO-EG [39]; **(b) **Nonoxide nanofluids: CNT-EG [8]; DNP-EG [40]; Graphene-EG [41]; Cu-EG [42].

The idea of nanofluid application originated from the fact that the thermal conductivity of a solid is much higher than that of a liquid. For example, the thermal conductivity of the most used conventional heat transfer fluid, water, is about 0.6 W/m · K at room temperature, while that of copper is higher than 400 W/m · K. Therefore, particle loading would be the chief factor that influences the thermal transport in nanofluids. As expected, the thermal conductivities of the nanofluids have been increased over that of the base fluid with the addition of a small amount of NPs. Figure 1 shows the enhanced thermal conductivity ratios of the nanofluids with NPs at different volume fractions [7,8,38-42]. (*k *- *k*_0_)/*k*_0 _and *φ *refer to the thermal conductivity enhancement ratio of nanofluids and the volume fraction of NPs, respectively, in this article. Figure 1a presents oxide nanofluids, while Figure 1b presents nonoxide nanofluids. The results show that all the nanofluids have noticeable higher thermal conductivities than the base fluid without NPs. In general, the thermal conductivity enhancement increases monotonously with the volume fraction. For the graphene nanofluid with a volume fraction of 0.05, the thermal conductivity can be enhanced by more than 60.0%. There is an approximate linear relationship between the thermal conductivity enhancement ratios and the volume fraction of graphene nanosheets. The nanofluids containing graphene nanosheets show larger thermal conductivity enhancement than those containing oxide NPs. It demonstrates that graphene nanosheet is a good additive to enhance the thermal conductivity of base fluid. However, the enhancement ratios of nanofluids containing graphene nanosheets are less than those of CNTs with the same loading. Many factors have direct influence on the thermal conductivity of the nanofluid. One of the important factors is the crystal structure of the inclusion in the nanofluid. Graphene is a one-atom-thick planar sheet of sp^2^-bonded carbon atoms that are densely packed in a honeycomb crystal lattice. The perfect structure of graphene is damaged when graphite is chemically oxidized by treatment with strong oxidants. There is no doubt that the high thermal conductivity is diminished by defects, and the defects have direct influence on the heat transport along the 2-D structure.

### Temperature

Some studies have demonstrated that the temperature has a great effect on the enhancement of the thermal conductivity for nanofluids. However, there is considerable disagreement in the literature with respect to the temperature dependence of their thermal conductivity. For example, Das et al. reported strong temperature-depended thermal conductivity for water-based Al_2_O_3 _and CuO nanofluids [43]. The thermal conductivity enhancements of nanofluids containing Bi_2_Te_3 _nanorods in FC72 and in oil had been experimentally found to decrease with increasing temperature [44]. Micael et al. measured the thermal conductivities of EG-based Al_2_O_3 _nanofluids at temperatures ranging from 298 to 411 K. A maximum in the thermal conductivity was observed at all mass fractions of NPs [45].

**Figure 2:**
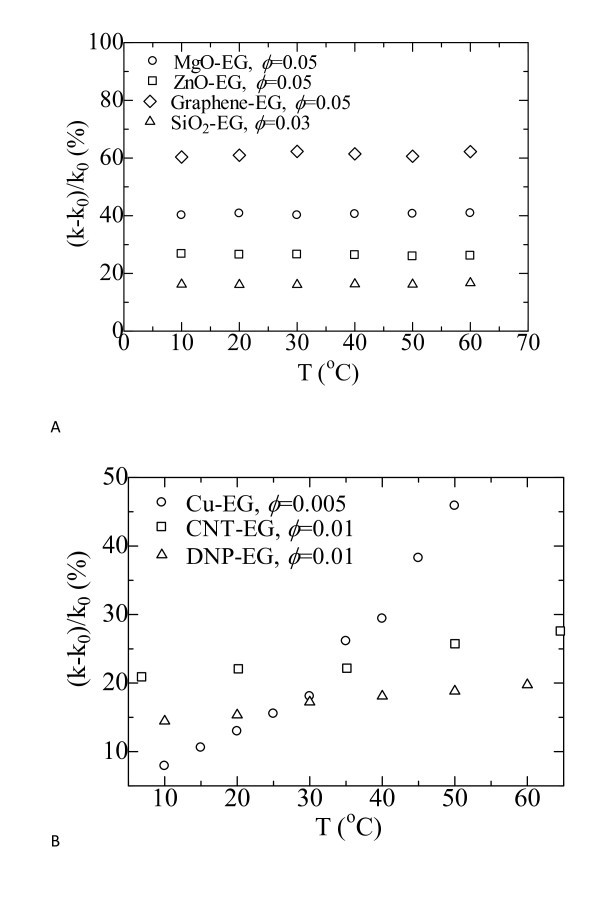
**Thermal conductivity enhancement varying with the tested temperatures**. **(a) **Oxide nanofluids: MgO-EG [38]; ZnO-EG [39]; Graphene-EG [41]; **(b) **Nonoxide nanofluids: Cu-EG [42]; CNT-EG [8]; DNP-EG [40].

Figure 2 shows our measured temperature-depended thermal conductivity enhancements of nanofluids [8,38-42]. For EG-based nanofluids containing MgO, ZnO, SiO_2_, and graphene NPs, the thermal conductivity enhancements almost remain constant when the tested temperature changes (see Figure 2a), which means that the thermal conductivity of the nanofluid tracks the thermal conductivities of the base liquid in the experimented temperature range of this study. The thermal conductivity enhancements of DW-EG-based nanofluids containing MgO, ZnO, SiO_2_, Al_2_O_3_, Fe_2_O_3_, TiO_2_, and graphene NPs also appear to have the same behavior. It was further found that kerosene-based Fe_3_O_4 _nanofluids presented temperature-independent thermal conductivity enhancements. Patel et al. [46] reported that the thermal conductivity enhancement ratios of Cu nanofluids are enhanced considerably when the temperature increases. The experimental results of this study shown in Figure 2b demonstrated similar tendency. At 10°C, the thermal conductivity enhancement of EG based Cu nanofluid with 0.5% nanoparticle loading is less than 15.0%. When the temperature is increased to 60°C, the enhancement reaches as large as 46.0%. Brownian motion of the NPs has been proposed as the dominant factor for this phenomenon. For the EG-based CNT nanofluids, cylindrical nanotubes with large aspect ratios were used as additions. The effect of Brownian motion will be negligible. Typical conduction-based models will give (*k *- *k*_0_)/*k*_0_, independent of the temperature. However, results shown in Figure 2b illustrate that (*k *- *k*_0_)/*k*_0 _increases, though not drastically, with the temperature. CNT aggregation kinetics may contribute to the observed differences [21]. It is worthy of bearing in mind that the temperatures of the base fluid and the nanofluid should be the same when compared with the thermal conductivities between them. Comparison of the thermal conductivities between the nanofluid at one temperature and the base at another one is meaningless.

### Base fluid

**Figure 3:**
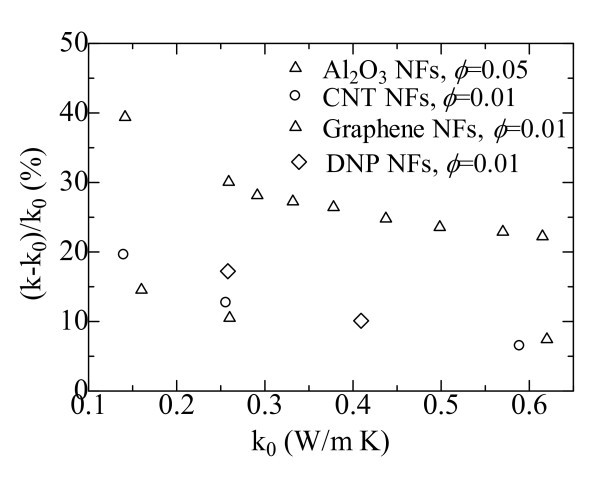
**Thermal conductivity enhancement ratios as a function of the thermal conductivities of the base fluids: Al**_**2**_**O**_**3 **_**NFs **[7]**; CNT NFs **[8]**; Graphene NFs **[41]**; DNP NFs **[40].

Figure 3 shows the relation between the enhanced thermal conductivity ratios of the nanofluids and the thermal conductivities of the base fluids [7,8,40,41]. It is clearly seen that no matter what kind of nanoparticle was used, the thermal conductivity enhancement decreases with an increase in the thermal conductivity of the base fluid. For pump oil (PO)-based Al_2_O_3 _nanofluid with 5.0% nanoparticle loading, the thermal conductivity can be enhanced by more than 38% compared to that of PO. When the base fluid is substituted with water, the thermal conductivity enhancement achieved is only about 22.0% [7]. A greater dramatic improvement in thermal conductivity of CNT nanofluid is seen for a base fluid with lower thermal conductivity. At 1.0% nanoparticle loading, the thermal conductivity enhancements are 19.6, 12.7, and 7.0% for CNT nanofluids in decene, EG, and DW, respectively. No matter what kind of base fluid is used, the thermal conductivity enhancement of CNT nanofluids is much higher than that for Al_2_O_3 _nanoparticle suspensions [8] at the same volume fraction. The reason would lie in the substantial difference in thermal conductivity and morphology between alumina nanoparticle and carbon nanotube.

### Particle size

**Figure 4:**
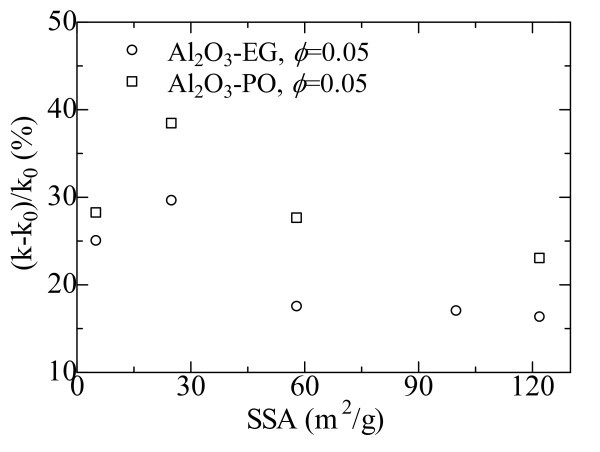
**Enhanced thermal conductivity ratios as a function of the SSAs: Al**_**2**_**O**_**3**_**-EG **[7]**; Al**_**2**_**O**_**3**_**-PO **[7].

Figure 4 presents the thermal conductivity enhancement of the nanofluids as a function of the specific surface area (SSA) of the suspended particles [7]. It is seen that the thermal conductivity enhancement increases first, and then decreases with an increase in the SSA, with the largest thermal conductivity at a particle SSA of 25 m^2 ^· g^-1^. We ascribe the thermal conductivity change behavior to twofold factors. First, as particle size decreases, the SSA of the particle increases proportionally. Heat transfer between the particle and the fluid takes place at the particle-fluid interface. Therefore, a dramatic enhancement in thermal conductivity is expected because a reduction in particle size can result in large interfacial area. Second, the mean free path in polycrystalline Al_2_O_3 _is estimated to be around 35 nm, which is comparable to the size of the particle that was used. The intrinsic thermal conductivity of nanosized Al_2_O_3 _particle may be reduced compared to that of bulk Al_2_O_3 _due to the scattering of the primary carriers of energy (phonon) at the particle boundary. It is expected that the suspension's thermal conductivity is reduced with an increase in the SSA. Therefore, for a suspension containing NPs at a particle size much different from the mean free path, the thermal conductivity increases when the particle size decreases because the first factor is dominant. However, when the size of the dispersed NPs is close to or smaller than the mean free path, the second factor will govern the mechanism of the thermal conductivity behavior of the suspension.

**Figure 5:**
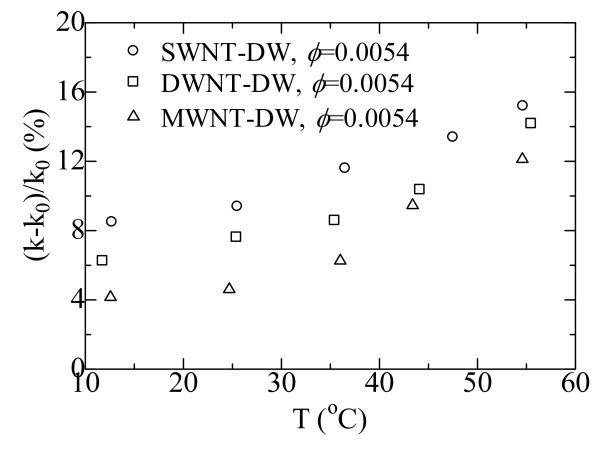
**Thermal conductivity enhancements of nanofluids containing CNTs with different sizes: SWNT-DW **[47]**; DWNT-DW **[47]**; MWNT-DW **[47].

Figure 5 depicts the thermal conductivity enhancements of nanofluids containing CNTs with different sizes [47]. The base fluid is DW, and the volume fraction of the CNTs is 0.0054. It is observed from Figure 5 that the thermal conductivity enhancements show differences among these three kinds of nanofluids containing SWNTs, DWNTs, and MWNTs as the volume fraction of CNTs is the same. Two influencing factors may be addressed. The first one is the intrinsic heat transfer performance of the CNTs. It is reported that the thermal conductivity of CNTs decreases with an increase in the number of the nanotube layer. The tendency of the thermal conductivity enhancement of the obtained CNT nanofluids accords with that of the heat transfer performance of the three kinds of CNTs. The second one is the alignment of the liquid molecules on the surface of CNTs. There are greater number of water molecules close to the surfaces of CNTs with smaller diameter due to the larger SSA if the volume fractions of CNTs are the same. These water molecules can form an interfacial layer structure on the CNT surfaces, increasing the thermal conductivity of the nanofluid [47].

### Pretreatment

In the preparation of nanofluids, solid additives are always subjected to various pretreatment procedures. The initial incentive is to tailor the surfaces of the NPs to enhance their dispersibility, thereby to enhance the stability of the nanofluids. The morphologies would be significantly changed when CNTs were subjected to chemical or mechanical treatments. Theoretical research into the thermal conductivity of composites containing cylindrical inclusions has demonstrated that the morphologies, including the aspect ratio, have influence on the effective thermal conductivity of the composites. Therefore, it can be expected that the thermal conductivity of CNT contained nanofluids would be affected by the pretreatment process.

**Figure 6:**
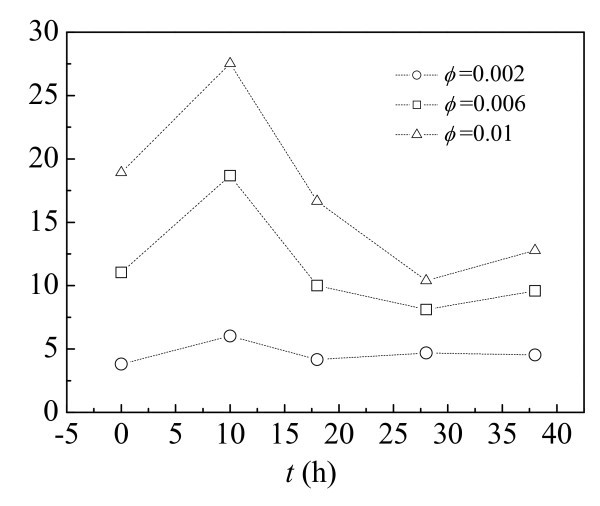
**Dependence of the thermal conductivity enhancement on the ball milling time of CNTs suspended in the nanofluids **[48].

Figure 6 shows the dependence of the thermal conductivity enhancement on the ball milling time of CNTs suspended in the nanofluids [48]. From theoretical prediction, the thermal conductivity of a composite increases with the aspect ratio of the included solid particles [49-51]. Intuition suggests that increasing the milling time should therefore decrease (*k *- *k*_0_)/*k*_0 _because of the reduced aspect ratio. Figure 6, however, shows clear peak and valley values in the thermal conductivity enhancement with respect to the milling time for all the studied CNT loadings. For nanofluid at a volume fraction of 0.01, the thermal conductivity enhancements present a peak value of 27.5% and a valley value of 10.4% when the milling times are 10 and 28 h, respectively. The maximal enhancement is intriguingly more than two and half times as the minimal one. Interestingly, when further increased the milling time from 28 to 38 h, (*k *- *k*_0_)/*k*_0 _increases from the valley value of 10.4 to 12.8%. Though the increment is not pronounced, it illustrates a difference in tendency from that in the milling time range from 10 to 28 h. Temperature-dependent thermal conductivity enhancement data further indicate that, at all the measured temperatures, nanofluid with CNTs milled for 10 h has the largest increment in thermal conductivity. Glory et al. [52] reported that the enhancement of the thermal conductivity noticeably increases when the nanotube aspect ratio increases. However, the thermal conductivity enhancement behavior of our CNT nanofluid is very different and cannot be explained only by the effect of the aspect ratio.

The above results suggest other dominant factors that have the influence over the thermal conductivity of the CNT nanofluids. The authors proposed that the nonstraightness and the aggregation would play significantly roles. As is known, the walls of CNTs have similar structure of graphene sheet, and the thermal conductivity of CNTs shows greatly anisotropic behavior. Heat transports substantially quicker through axial direction than through radial direction [53]. For a nonstraight CNT, the high thermal anisotropy of CNTs induces a unique property that individual CNTs are nearly perfect one-dimensional thermal passages with negligibly small heat flux losses during long distance heat conductions [54]. For a nonstraight CNT with length *L *under a two-end temperature difference, the heat flux *q *goes through a curled passage. This CNT can be regarded as an equivalent straight thermal passage with a distance of *L*^e^. The same heat flux *q *is conducted between the two ends of this straight passage. Obviously, the equivalent length *L*^e ^depends on the curvature of the actual nanotube in the nanofluid. A concept, straightness ratio *η *(*η = L*^e^/*L*), can be adopted to describe the straightness of a curled CNT. The lowest straightness ratio arises when a suspended nanotube forms ring closure [55].

When subjected to ball milling, CNTs were broken and cut short with appropriate average length. The straightness ratio was significantly increased and heat transports more effectively through the CNTs and across the interfaces between the CNT tips and the base fluid, resulting in the highest thermal conductivity enhancement in a nanofluid containing CNTs milled for 10 h. For nanofluids containing relatively straight nanotubes, the influence of the aspect ratio will surpass that of straightness ratio. Therefore, by further treatment on nanotubes with relatively high straightness ratio, the excessive deterioration of the aspect ratio would decrease the thermal conductivity of nanofluids, causing (*k *- *k*_0_)/*k*_0 _decrease from 10 to 28 h. Recent theoretical analysis has revealed that the aggregation of nanoparticle plays a significant role in deciding (*k *- *k*_0_)/*k*_0 _[21]. Percolation effects in the aggregates, as highly conducting nanotubes touch each other in the aggregate, help in increasing the thermal conductivity. Our experiments demonstrate that aggregates are the dominant appearance of CNTs when the ball-milling time is increased to 38 h. The aggregation accounts for the increment of thermal conductivity enhancement when the ball-milling time is increased from 28 to 38 h. This result implies that the positive influence of the aggregation surpasses the negative influence of the aspect ratio deterioration.

### pH value

**Figure 7:**
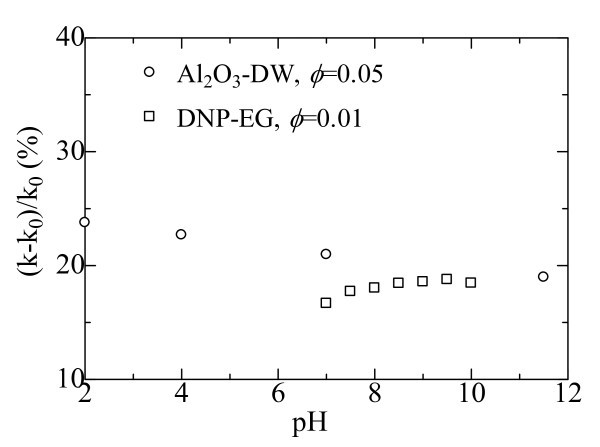
**Thermal conductivity enhancement ratios at different pH values: Al**_**2**_**O**_**3**_**-DW **[7]**; DNP-EG **[40].

For some nanofluids, the pH values of the suspensions have direct effects on the thermal conductivity enhancement. Figure 7 presents the thermal conductivity enhancement ratios at different pH values [7,40]. The results show that the enhanced thermal conductivity increases with an increase in the difference between the pH value of aqueous suspension and the isoelectric point of Al_2_O_3 _particle [7]. When the NPs are dispersed into a base fluid, the overall behavior of the particle-fluid interaction depends on the properties of the particle surface. For Al_2_O_3 _particles, the isoelectric point (pH_iep_) is determined to be 9.2, i.e., the repulsive forces among Al_2_O_3 _particles is zero, and Al_2_O_3 _particles will coagulate together under this pH value. Therefore, when pH value is equal or close to 9.2, Al_2_O_3 _particle suspension is unstable according to DLVO theory [56]. The hydration forces among particles increase with the increasing difference of the pH value of a suspension from the pH_iep_, which results in the enhanced mobility of NPs in the suspension. The microscopic motions of the particles cause micro-convection that enhances the heat transport process. Wensel's study showed that the thermal conductivity of nanofluids containing oxide NPs and CNTs with very low percentage loading decreased when the pH value is shifted from 7 to 11.45 under the influence of a strong outside magnetic field [14].

For DNP-EG nanofluids, it is observed from Figure 7 that the thermal conductivity enhancement increases with pH values in the range of 7.0-8.0. When pH value is above 8.0, there is no obvious relationship between pH value and the thermal conductivity enhancement. In our opinion, the influence of pH value on thermal conductivity is that pH value has a direct effect on the stability of nanofluids. When pH value is below 8.5, the suspension is not very stable, and DNPs are easy to form aggregations. The alkalinity of the solution is helpful to the dispersion and the stability of the nanofluids. In order to verify the above statement, the influence of settlement time on the thermal conductivity enhancement was further investigated. It is found that the thermal conductivity enhancement decreases with elapsed time for DNP-EG nanofluid when pH is 7.0. However, for the stable DNP-EG nanofluids with pH of 8.5, there is no obvious thermal conductivity decrease for 6 months [40].

### Surfactant addition

Surfactant addition is an effective way to enhance the stability of nanofluids. Kim's study revealed that the thermal conductivity decreased rapidly for the instable nanofluids without surfactants after preparation. However, no obvious changes in the thermal conductivity of the nanofluids with sodium dodecyl sulfate (SDS) as surfactant were observed even after 5-h settlement [57]. Assael et al. investigated the thermal conductivities of the aqueous suspension of CNTs. When Sodium dodecyl sulfate (SDS) was employed as the dispersant, the maximum thermal conductivity enhancement obtained was 38.0% for a nanofluid with 0.6 vol% CNT loadings [58]. When the surfactant is substituted with hexadecyltrimethyl ammonium bromide (CTAB), the maximum thermal conductivity enhancement obtained was 34.0% for same fraction of CNT loading [26]. Liu et al. reported that the thermal conductivity of carbon nanotube-synthetic engine oil suspensions is higher compared with that of same suspensions without the addition of surfactant. The presence of surfactant as stabilizer has positive effect on the carbon nanotube-synthetic engine oil suspensions [59].

**Figure 8:**
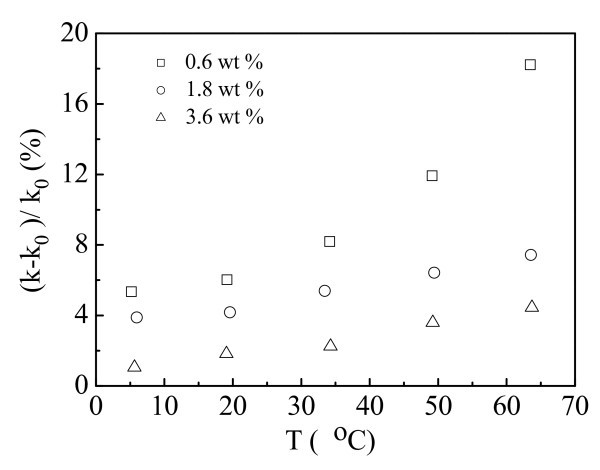
**Thermal conductivity enhancement ratios with different surfactant concentrations**.

We used cationic gemini surfactants (12-3(4,6)-12,2Br^-1^) to stabilize water-based MWNT nanofluids. These surfactants were prepared following the process described in [60]. Figure 8 presents the thermal conductivity enhancement ratios of the CNT-contained nanofluids with different surfactant concentrations. The volume fraction of the dispersed CNTs is 0.1%. The critical micelle concentration of 12-3-12, 2Br^-1 ^is reported as 9.6 ± 0.3 × 10^-4 ^mol/l [61]. Ten times critical micelle concentration of 12-3-12, 2Br^-1 ^is 0.6 wt%. Solutions of 12-3-12, 2Br^-1 ^with different concentrations (0.6, 1.8, and 3.6 wt% at room temperature) were selected to prepare CNT nanofluids. It is observed that at all the measured temperatures the thermal conductivity enhancement decreases with the surfactant addition. The surfactant added in the nanofluids acts as stabilizer which improves the stability of the CNT nanofluids. However, excess surfactant addition might hinder the improvement of the thermal conductivity enhancement of the nanofluids.

**Figure 9:**
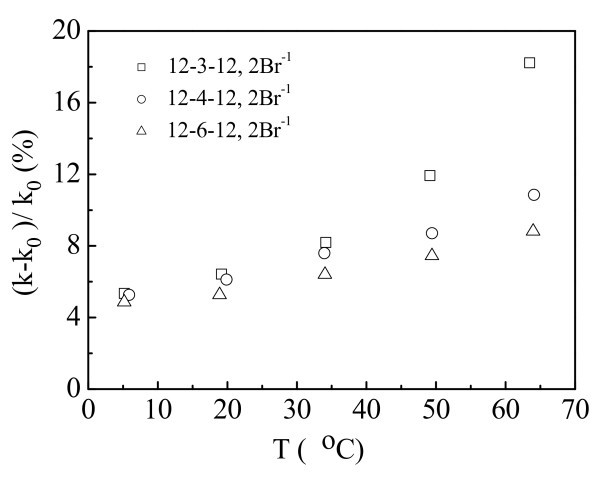
**Effect of surfactant structures on the thermal conductivity enhancement ratio**.

The effect of the structures of cationic gemini surfactant molecules on the thermal conductivity enhancement is shown in Figure 9. The fractions of the dispersed CNTs and the cationic gemini surfactants is 0.1 vol% and 0.6 wt%, respectively. The spacer chain length of the cationic gemini surfactant increase from 3 methylenes to 6 methylenes. It is seen that the thermal conductivity enhancement ratio increases with the decrease of spacer chain length of cationic gemini surfactant. Zeta potential analysis indicates that the CNT nanofluids stabilized by gemini surfactant with short spacer chain length have better stabilities. Increase of spacer chain length of surfactant might give rise to sediments of CNTs in the nanofluids, resulting in the decrease of thermal conductivity enhancement of the nanofluids.

## Conclusions

Nanofluids have great potential for heat transfer enhancement and are highly suited to application in practical heat transfer processes. This provides promising ways for engineers to develop highly compact and effective heat transfer equipments. More and more researchers have paid their attention to this exciting field. When addressing the thermal conductivity of nanofluids, it is foremost important to guarantee the accuracy in the measurement of the thermal conductivity of nanofluids. Two aspects should be considered. The first one is to prepare homogeneous and long-term stable nanofluids. The second one is to keep the initial equilibrium before measuring the thermal conductivity. In general, the thermal conductivity enhancement increases monotonously with the particle loading. The effect of temperature on the thermal conductivity enhancement ratio is somewhat different for different nanofluids. It is very important to note that the temperatures of the base fluid and the nanofluid should be the same while comparing the thermal conductivities between them. With an increase in the thermal conductivity of the base fluid, the thermal conductivity enhancement ratio decreases. Considering the effect of the size of the inclusion, there exists an optimal value for alumina nanofluids, while for the CNT nanofluid, the thermal conductivity increases with a decrease of the average diameter of the included CNTs. The thermal characteristics of nanofluids might be manipulated by means of controlling the morphology of the inclusions, which also provide a promising way to conduct investigation on the mechanism of heat transfer in nanofluids. The additives like acid, base, or surfactant play considerable roles on the thermal conductivity enhancement of nanofluids.

## Abbreviations

CNTs: carbon nanotubes; DNPs: diamond NPs; DW: deionized water; DWNTs: double-walled CNTs; EG: ethylene glycol; KOH: potassium hydroxide; LP: liquid paraffin; MWNTs: multi-walled CNTs; NPs: nanoparticles; PVP: poly(vinylpyrrolidone); SDS: sodium dodecyl sulfate; SHW: short hot wire; SSA: specific surface area; SWNTs: single-walled CNTs; THW: transient hot wire; TCNTs: treated CNTs.

## Competing interests

The authors declare that they have no competing interests.

## Authors' contributions

HQ supervised and participated all the studies. He wrote this paper. WY carried out the studies on the nanofluids containing copper nanoparticles, graphene, diamond nanoparticles, and several kinds of oxide nanoparticles. YL carried out the studies on the nanofluids containing other oxide nanoparticles. LF carried out the studies on the nanofluids containing carbon nanotubes.
